# Pediatric acute lymphoblastic leukemia relapse and prognosis: key predictors and therapeutic implications

**DOI:** 10.3389/fped.2025.1710578

**Published:** 2025-12-18

**Authors:** XiaoYan Chen, LingLing Wu, CaiYun Kuang, JiaYi Wang, WenGe Hao, Hua Jiang, WeiNa Zhang

**Affiliations:** Department of Hematology and Oncology, Guangzhou Women and Children’s Medical Center, Guangzhou Medical University, Guangzhou, China

**Keywords:** acute lymphoblastic leukemia, immunothearpy, pediatric, prognosis, relapse

## Abstract

**Background:**

Pediatric acute lymphoblastic leukemia (ALL), the most common childhood malignancy, achieves >95% 5-year survival with risk-adapted therapies. Nonetheless, 10%–15% of patients experience relapse, with post-relapse survival <50%. Challenges remain in optimizing minimal residual disease (MRD)-guided strategies and salvage therapies in ALL.

**Aims:**

This study aimed to identify relapse predictors and assess post-relapse outcomes among 436 pediatric ALL patients treated according to the CCCG-ALL-2015 protocol.

**Results:**

Of the 436 enrolled patients (median age: 3.9 years; 92.4% B-ALL), sixty-four patients (14.7%) relapsed, predominantly with isolated bone marrow involvement (71.9%). Independent predictors included thrombocytopenia at diagnosis (OR = 2.09, *P* = 0.037), *BCR::ABL1*(+) (OR = 3.85, *P* = 0.024), and positive MRD on day 19 (OR = 2.09) and day 46 (OR = 5.73, *P* < 0.001) of induction therapy. Post-relapse, isolated extramedullary cases showed higher OS (100% vs. 72.9%, *P* = 0.078) than bone marrow relapses. HSCT significantly improved OS in bone marrow relapse comparing to patients treated with chemotherapy or CAR-T alone (82.6% vs. 38.1%, *P* = 0.027).

**Conclusion:**

Thrombocytopenia at diagnosis, *BCR::ABL1*(+), and persistent MRD are critical relapse predictors. HSCT remains pivotal for bone marrow relapse. Incorporating platelet counts into risk stratification and optimizing MRD-guided bridging therapies may enhance outcome. Future research should prioritize thrombocytopenia mechanisms and HSCT preconditioning strategies.

## 1 Introduction

Acute lymphoblastic leukemia (ALL), the most common pediatric malignancy, has undergone transformative improvements in survival through risk-stratified treatment protocols. Current therapeutic strategies achieve 5-year event-free survival (EFS) rates of over 85% and overall survival (OS) rates approaching 95% (1–3). Nevertheless, approximately 10% of pediatric ALL patients experience relapse, and post-relapse OS plummets to below 50% (4). Identifying risk factors associated with relapse and reducing relapse incidence remain crucial priorities for optimizing treatment frameworks. Present strategies focus on refining risk stratification, enhancing minimal residual disease (MRD)-guided interventions, and incorporating novel targeted therapies to address these challenges.

To examine relapse dynamics, we performed a retrospective cohort study of pediatric ALL cases. This study sought to: (1) compare baseline clinical, biological, and treatment-response profiles between relapsed and non-relapsed patients; (2) identify novel prognostic markers using multivariate regression analysis; and (3) assess outcomes across subgroups stratified by relapse timing, site, and response to salvage therapy. By integrating molecular profiling, immunophenotypic data, and MRD monitoring, this research aims to refine risk classification systems, suggest tailored therapeutic strategies, and advance precision medicine for relapsed disease. The findings could help optimize relapse-prevention algorithms and improve survival in high-risk populations.

## 2 Materials and methods

### 2.1 Study cohort

This retrospective study analyzed clinical data from pediatric ALL patients treated according to the CCCG-ALL-2015 protocol (5) (Clinical Trial Registry: ChiCTR-IPR-14005706) at Guangzhou Women and Children's Medical Center between March 2015 and December 2019. The study protocol received ethical approval from the institutional review board (IRB Approval No.: 2025020936), with written informed consent obtained from all legal guardians.

Inclusion criteria: (1) Age ≥1 month and <18 years at diagnosis. (2) *de novo* ALL confirmed by bone marrow morphology and immunophenotyping. (3) Standardized frontline therapy per the CCCG-ALL-2015 protocol.

Exclusion criteria: (1) Prior treatment for ALL. (2) Immediate treatment abandonment post-diagnosis. (3) Non-protocol-compliant therapy. (4) Failure to achieve remission or pre-remission mortality.Treatment protocol: Treatment consisted of a window period, induction, consolidation, and continuation therapy. Risk stratification incorporated age, initial white blood cell (WBC) count, immunophenotype (B- or T-lineage), cytogenetic/molecular profiles (e.g., *BCR::ABL1*, *ETV6::RUNX1*), and MRD levels monitored via multiparameter flow cytometry. For relapsed ALL, salvage strategies (non-randomized) included re-induction chemotherapy, radiotherapy, chimeric antigen receptor T-cell (CAR-T) therapy, or allogeneic hematopoietic stem cell transplantation (HSCT), tailored according to relapse site and patient condition. HSCT was recommended for all patients with bone marrow relapsed diseases or those classified as high-risk group. It should be note that the use of CAR-T therapy or HSCT in this study was influenced by economic factors and treatment accessibility, which may introduce selection bias and limit the generalizability of salvage therapy outcomes.

The treatment regimen of CCCG-2015 protocol is summarized in Supplementary Table S1. Patients with lumbar puncture injury at diagnosis received additional spinal punctures with intrathecal methotrexate and intensified methotrexate dosing for testicular involvement—consistent with intermediate-risk escalation—even in the absence of other risk factors, along with subsequent chemotherapy intensification.

Of the initial cohort, five patients who failed to achieve complete remission after first induction therapy and died from disease-related causes were excluded. The remaining 436 patients, all of whom achieved complete remission (CR) after first induction therapy, were included in the analysis. The CR rate after induction in the initial screened cohort was 98.9%.

### 2.2 Follow-up methodology

Follow-up data were collected from electronic medical records, outpatient visits, and structured telephone interviews. Outcomes assessment included relapse events, mortality, and severe treatment-related complications. The observation period continued until December 12, 2024, with a median follow-up duration of 81 months (range: 11–117 months). OS was defined as the time from diagnosis to death or the last follow-up. Relapse-free survival (RFS) was calculated from diagnosis until the first relapse or the last follow-up for patients who remained event-free.

### 2.3 Definitions

#### 2.3.1 Relapse classification

Relapses were categorized based on timing and anatomical site as follows: Temporal Classification: Very early relapse: <18 months post-diagnosis. Early relapse: 18–36 months post-diagnosis. Late relapse: >36 months post-diagnosis. Anatomic Classification: Isolated bone marrow relapse: ≥20% blasts in bone marrow aspirate without extramedullary involvement. Isolated extramedullary relapse: Recurrence in central nervous system (CNS), testes, or other sites, confirmed by histopathology or cerebrospinal fluid (CSF) analysis. Combined relapse: Concurrent marrow and extramedullary disease.

#### 2.3.2 CNS involvement criteria

CNS-3: CSF WBC ≥5/*μ*L with blasts on cytocentrifugation, neurologic deficits, or imaging evidence of infiltration. CNS-2: CSF WBC <5/μL with blasts. CNS-1: Absence of blasts. Traumatic lumbar puncture: Blood-contaminated CSF (red blood cell ≥ 10/μL).

#### 2.3.3 MRD definitions

MRD positivity: ≥0.01% leukemic blasts detected by flow cytometry with bone marrow samples on day 19 (D19-MRD) or 46 (D46-MRD) of induction. MRD negativity: <0.01% blasts detected by flow cytometry on day 19 or 46 of induction. Time to MRD negativity: Duration from diagnosis to first MRD-negative assessment.

#### 2.3.4 Risk stratification

Patients with B-ALL aged 1 to <10 years, and initial leukocyte count <50 × 10^9^/L, and the presence of favorable genetic features such as hyperdiploidy (>50 chromosomes), or *ETV6:: RUNX1* fusion and without CNS3 status, no testicular leukemia, MRD <1% on day 19 of induction, and MRD <0.01% on day 46 of induction were classified as having low-risk disease. Patients with MRD ≥1% (or ≥5% blasts morphologically without suitable markers for MRD) in bone marrow on day 46 of induction and infants under 6 months of age with *KMT2A* rearrangement and initial leukocyte count ≥300 × 10^9^/L were considered to have high-risk ALL. The remaining cases were classified as intermediate-risk ALL.

### 2.4 Statistical analysis

Statistical analyses were conducted using SPSS 27.0 and GraphPad Prism 10.0. Continuous variables with non-normally distribution were summarized as medians (ranges), while categorical variables were expressed as frequencies (percentages). Group comparisons utilized *Student's t-test* (normally distributed data), *Mann–Whitney U*-test (non-parametric data), or *χ^2^* test (categorical variables).

Univariate and multivariate logistic regression models were employed to identified risk factors associated with relapse. Survival analyses employed Kaplan–Meier curves with log-rank testing for intergroup comparisons. A two-sided *P*-value <0.05 was considered statistically significant.

## 3 Results

### 3.1 Clinical characteristics of the study cohort

The study included 436 pediatric ALL patients with a median age of 3.9 years (range: 1 month–17 years) and a male-to-female ratio of 1.53:1. Immunophenotypic analysis classified 403 (92.4%) cases as B-cell ALL (B-ALL) and 33 (7.6%) as T-cell ALL (T-ALL). Risk stratification classified 217 patients (49.8%) as low-risk (LR), 214 (49.1%) as intermediate-risk (IR), and 5 (1.1%) as high-risk (HR). The different states of the CNS status at diagnosis are as follows:416 patients (95.4%) were classified as CNS1, while 20 (4.6%) exhibited CNS-2, CNS-3, or traumatic lumbar puncture. Eight (1.8%) patients presented with testicular leukemia. Molecular profiling revealed recurrent genetic alterations: *ETV6::RUNX1* (*n* = 89, 20.4%), *TCF3::PBX1* (*n* = 21, 4.8%), *BCR::ABL1* (*n* = 15, 3.4%), and *KMT2A* rearrangements (*n* = 7, 1.6%). At final follow-up, 372 (85.3%) patients remained in continuous complete remission, while 64 (14.7%) experienced relapse. Among all the patients, the RFS was 85.1 ± 1.7% and the OS was 94.9 ± 1.1%. No treatment-related deaths occurred prior to relapse. And none of the high-risk patients underwent HSCT after achieving first CR which was decided by economic factors and treatment accessibility of parents.

Among the 64 relapsed patients, the median time to relapse was 33 months (range: 2–64 months). Relapse occurred during treatment in 29 patients, within one year after treatment cessation in 21 patients, and one year or more after treatment completion in 14 patients. Temporal relapse distribution included very early relapse (*n* = 13, 20.3%), early relapse(*n* = 28, 43.8%), and late relapse (*n* = 23, 35.9%). Anatomic relapse patterns comprised isolated bone marrow (*n* = 46, 71.9%), isolated extramedullary (*n* = 12, 18.8%), and combined relapse (*n* = 6, 9.4%). Molecular profiling of relapsed cases revealed *ETV6::RUNX1* (*n* = 11), *BCR::ABL1* (*n* = 6), *TCF3::PBX1* (*n* = 1), and *KMT2A* rearrangements (*n* = 1). No significant associations were observed between molecular subtypes and relapse timing or site (*P* > 0.05).

### 3.2 Comparative analysis of relapsed vs. sustained remission cohorts and risk factor assessment

The relapsed cohort exhibited distinct clinical characteristics compared to the sustained remission group, including higher proportions of intermediate- or high-risk stratification, thrombocytopenia at diagnosis, and *BCR::ABL1*(+). Relapsed patients exhibited higher rates of MRD positivity during induction therapy and a prolonged time to MRD negativity. The detailed comparison of the characteristics between two groups is shown in Table 1 as follows.

**Table 1:** Comparison of clinical characteristics between sustained remission and relapsed groups in pediatric acute lymphoblastic leukemia.

Groups	CR group（*n* = 372)	Relapse group (*n* = 64)	Statistical value(*χ^2^*or *U*)	*P*-value
Gender			1.383	0.269
Male	221（59.4%）	43 (67.2%)		
Female	151 (40.6%)	21 (32.8%)		
Age(years old)			2.278	0.203
<10	340 (91.4%)	62 (96.9%)		
≥10	32 (8.6%)	2 (3.1%)		
Immunophenotype				
B	341 (91.7%)	62 (96.9%)	2.117	0.201
T	31 (8.3%)	2 (3.1%)		
Final risk group			10.323	0.006
LR	196 (52.7%)	21 (32.8%)		
IR	173 (46.5%)	41 (64.1%)		
HR	3 (0.8%)	2 (3.1%)		
CNS status			3.595	0.166
CNS1	355 (95.4%)	61 (95.3%)		
CNS3	7 (1.9%)	3 (4.7%)		
Lumbar puncture injury	10 (2.7%)	0 (0.0%)		
testicular leukemia			1.402	0.236
Yes	8 (2.2%)	0 (0.0%)		
No	364 (97.8%)	64 (100.0%)		
WBC(×10^9^/L)			0.027	0.869
>50	78 (18.9%)	14 (21.(%)		
≤50	294 (79.0%)	50 (78.1%)		
Hb(g/L)			0.879	0.435
>90	276 (74.2%)	51 (79.7%)		
≤90	96 (25.8%)	13 (83.2%)		
PLT(×10^9^/L)			5.68	0.023
>20	322 (86.6%)	48 (75.0%)		
≤20	50 (13.4%)	16 (25.0%)		
*ETV6::RUNX1*			0.48	0.615
Yes	78 (21.0%)	11 (17.2%)		
No	294 (79.0%)	53 (82.8%)		
*TCF::PBX1*			1.732	0.338
Yes	20 (5.4%)	1 (1.6%)		
No	352 (94.6%)	63 (98.4%)		
*BCR::ABL1*			7.953	0.014
Yes	9 (2.4%)	6 (9.4%)		
No	363 (97.6%)	58 (90.6%)		
*KMT2Ar*			0.001	1.000
Yes	6 (1.6%)	1 (1.6%)		
No	366 (98.4%)	63 (98.4%)		
D19-MRD			13.338	<0.001
Positive	219 (58.9%)	53 (82.8%)		
Negative	153 (41.1%)	11 (17.2%)		
D46-MRD			47.598	<0.001
Positive	30 (8.1%)	39 (60.9%)		
Negative	342 (91.9%)	25 (39.1%)		
Time to MRD negativity (days)			9.102	0.003
Medium time	50.5	57		

**Figure 1:**
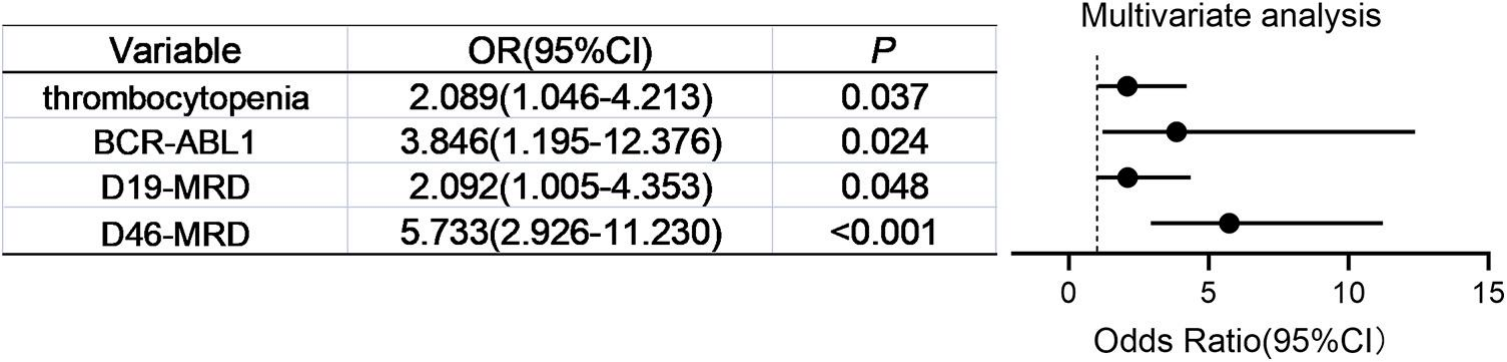
Multivariate analysis of independent relapse risk factors for pediatric ALL.

Multivariate logistic regression identified four independent relapse risk factors: thrombocytopenia at diagnosis [OR 2.089 (95% CI, 1.046–4.213), *P* = 0.037], *BCR::ABL1*(+) ALL [OR 3.846 (95% CI, 1.195–12.376), *P* = 0.024], positive D19-MRD [OR 2.092 (95% CI, 1.005–4.353), *P* = 0.048], and positive D46-MRD [OR 5.733 (95% CI, 2.926–11.230), *P* < 0.001] (Figure 1).

Subgroup analysis of *BCR::ABL1*(+) patients (*n* = 15) revealed divergent outcomes by tyrosine kinase inhibitor therapy. The imatinib cohort (*n* = 3) experienced universal bone marrow relapse (RFS 0.00 ± 0.0%) and poor OS (33.3 ± 27.2%). In contrast, those receiving dasatinib(*n* = 12) exhibited significantly superior RFS (72.9 ± 13.5%, *χ^2^* = 18.836, *P* < 0.001) and OS (83.3 ± 15.2%, *χ^2^* = 5.663, *P* = 0.017). As for patients with thrombocytopenia at diagnosis, further analysis of the clinical characteristics revealed that among the 16 relapsed cases, the majority also presented with concurrent leukocytosis and anemia but no significant correlation was observed with high MRD levels or specific fusion gene phenotypes. Notably, bone marrow relapse was particularly common in this group (13/16 cases). Further stratification by immunophenotype revealed that among B-ALL patients, those with thrombocytopenia had significantly inferior RFS compared to those without (74.9 ± 5.4% vs. 86.2 ± 1.9%, *χ*^2^ = 5.667, *P* = 0.017). In contrast, no such association was observed in the T-ALL cohort. These results suggest that thrombocytopenia may serve as a potential prognostic factor for refining risk stratification in B-ALL, but not in T-ALL.

### 3.3 Survival analysis of relapsed patients

Among the sixty-four relapsed patients, fourteen patients were excluded due to incomplete treatment records, and ten discontinued therapy, leaving 40 patients for survival analysis.

Extramedullary relapse occurred in ten cases, including seven cases of testicular leukemia, two cases of central nervous system leukemia (CNSL), and one case of relapse in the left renal region. Among patients with bone marrow involvement, twenty-six cases had isolated bone marrow relapse, two cases had combined bone marrow and testicular leukemia relapse, and two cases were combined bone marrow and CNSL relapse.

Post-Relapse Treatment Choices: For extramedullary relapse cases, four patients received chemotherapy, three underwent CAR-T therapy alone, one received CAR-T followed by chemotherapy, and one underwent HSCT. The patient with renal relapse received chemotherapy and radiotherapy followed by HSCT and remained disease-free at the follow-up date. Among the bone marrow relapse cases, two patients received CAR-T therapy alone, five patients received chemotherapy alone, and twenty-three patients underwent HSCT. Based on the timing of relapse, eight cases were classified as very early relapse, twenty-one cases as early relapse, and eleven cases as late relapse.

A total of eleven patients received CAR-T therapy and within which, two cases were treated at external institutions with unavailable details. Among the remaining nine cases, three cases received CD19-specific CAR with 4-1BB costimulatory domain, four received CD19-specific CAR with CD28 costimulatory domain, and two received CD19 and CD22-specific CAR with CD28 costimulatory domain.

**Figure 2:**
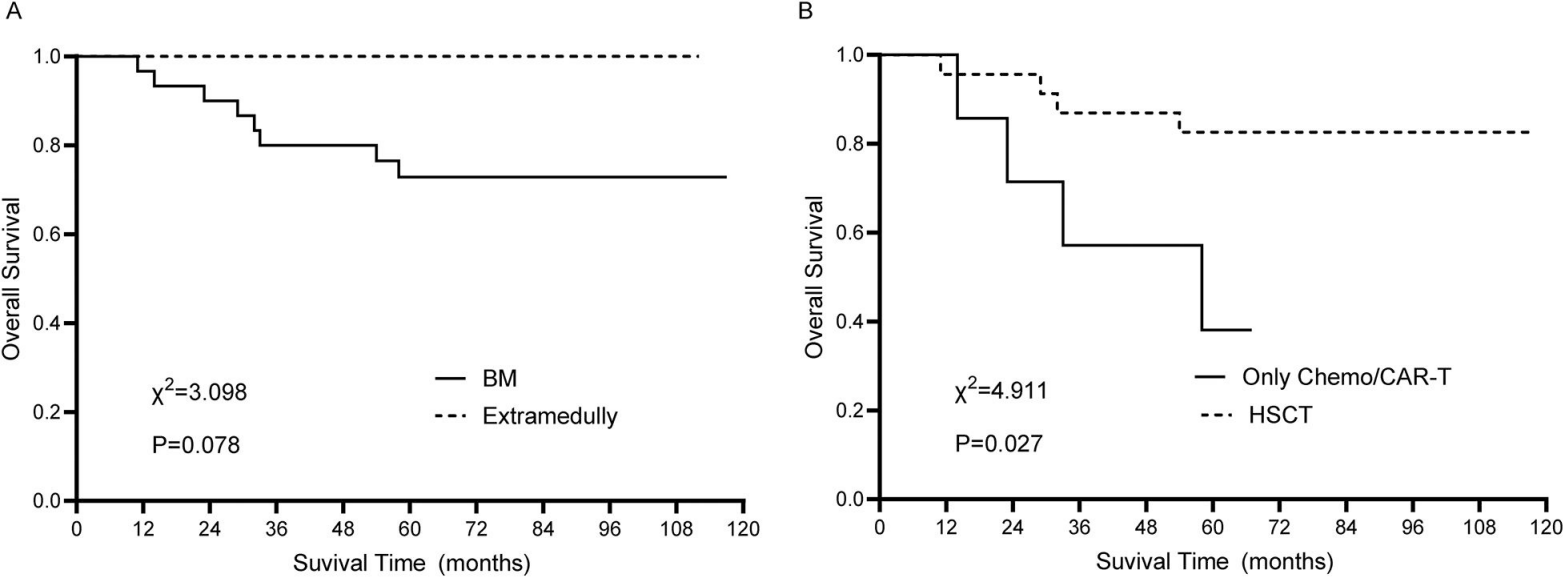
Survival analysis of relapse ALL patients. **(A)** Comparison between isolated extramedullary relapse group and bone marrow relapse group. **(B)** Comparison between chemotherapy or CAR-T alone group and HSCT group in bone marrow relapse patients.

Analysis of survival of relapse patients: Post-relapse mortality occurred in eight cases, including six deaths from disease progression and two treatment-related deaths. Survival analysis revealed no significant difference in OS among patients with different relapse timings (75.0 ± 15.3% vs. 76.2 ± 16.3% vs. 90.9 ± 8.7%, *χ^2^* = 1.330, *P* = 0.514). Patients with isolated extramedullary relapse showed better OS compared to those with bone marrow relapse, though this difference was not statistically significant (100.0 ± 0.0% vs. 72.9 ± 8.2%, *χ^2^* = 3.098, *P* = 0.078, Figure 2A). Among patients with bone marrow relapse, those treated with chemotherapy or CAR-T alone had significantly inferior OS compared to those undergoing HSCT (38.1 ± 19.9% vs. 82.6 ± 7.9%, *χ^2^* = 4.911, *P* = 0.027, Figure 2B).

## 4 Discussion

The evolution of risk-stratified chemotherapy has transformed pediatric ALL into a disease with OS rates exceeding 90%. Nevertheless, 15%–20% of patients experience relapse, with 5-year post-relapse OS remaining dismal (6). Our retrospective analysis identifies thrombocytopenia at diagnosis, *BCR::ABL1*(+), and suboptimal early treatment response [persistent minimal residual disease (MRD)] as independent predictors of relapse. Furthermore, we showed that post-relapse therapeutic strategies, particularly HSCT for bone marrow relapse, significantly affect survival outcomes. These findings highlighted the importance of integrating baseline biomarkers, dynamic MRD monitoring, and personalized salvage therapies to reduce relapse risk and enhance long-term survival.

Extensive studies have established several relapse-associated risk factors in pediatric ALL, including age extremes (infancy or >10 years), hyperleukocytosis (WBC >50 × 10⁹/L), CNS involvement, high post-induction MRD (≥0.01%), and adverse genetic abnormalities such as *KMT2A* rearrangements or *BCR::ABL1*. MRD during induction therapy, reflecting early chemosensitivity, is widely recognized as a critical prognostic factor. Current risk stratification in clinical protocols integrate clinical features, molecular genetics, and MRD levels (7–9). However, the optimal timing and threshold for MRD assessment remain subjects of ongoing debate. Most studies indicated that post-induction MRD exceeding 0.01% is associated with high-risk disease (10–12). Consistent with these reports, our study identified that positive MRD level at the end of induction therapy (day 46) and during induction therapy (day 19) are significant risk factors for relapse. Intensifying treatment for patients with persistently MRD positivity has been shown to improve outcomes (13), highlighting the clinical utility of MRD-based risk stratification in optimizing therapeutic strategies.

Beyond MRD, genetic subtypes play a critical role in ALL prognosis. Genetic abnormalities such as *TCF3::PBX1*, *BCR::ABL1*, *PAX5* mutations, and *IKZF1* deletions are associated with unfavorable outcomes, while hyperdiploidy and *ETV6::RUNX1* fusion gene are linked to more favorable prognosis (14). Our study confirmed that *BCR::ABL1*(+) patients have a higher relapse risk, while *TCF3::PBX1*(+) did not show a significant impact. Further analysis of *BCR::ABL1*(+) patients revealed that those treated with imatinib had inferior outcomes compared to those treated with dasatinib, consistent with findings from a previous collaborative study (15) but baseds on the small-scal research with a bias. However, some studies suggest comparable efficacy between dasatinib and imatinib, and despite the use of targeted therapy combined with chemotherapy, *BCR::ABL1*(+) patients exhibit inferior OS and EFS compared to *BCR::ABL1*(-) patient (16), indicating a need for further optimization of treatment strategies for this subgroup.

In addition to leukemia cell characteristics, normal hematopoietic recovery also influences prognosis in ALL. Platelet levels, reflecting the recovery of normal hematopoietic clones, have been shown to correlate with clinical outcomes. Slower and lower platelet recovery during or after induction therapy is associated with an unfavorable prognosis (17–19). Platelet recovery post-induction therapy may reflect the restoration of normal hematopoiesis after leukemia cell clearance, with slower recovery indicating an elevated risk of relapse. Furthermore, thrombocytopenia at diagnosis have been associated with increased relapse or mortality risk in pediatric ALL (20). Analysis of a population of 1545 children treated by the French Acute Lymphoblastic Leukemia Group and our study similarly found that patients with platelet counts below 20 × 10⁹/L at diagnosis of B-ALL had a higher relapse risk. Therefore, incorporating platelet levels at diagnosis into risk stratification, alongside MRD and molecular genetics, may further refine risk assessment and optimize treatment strategies. However, given the retrospective design of our study and continuous evolution of treatment protocols, the independent prognostic value of thrombocytopenia warrants prospective validation before it can be reliably integrated into clinical decision-making, particularly when considering major therapeutic interventions such as HSCT.

Beyond identifying risk factors for relapse, post-relapse treatment and outcomes represent another critical dimension of ALL research. Multiple factors such as age, timing of relapse, site of relapse, and high-risk genetic alterations significantly influence post-relapse survival (21). Our study found that patients with bone marrow relapse had worse outcomes compared to those with isolated extramedullary relapse. However, no significant differences in survival were observed based on relapse timing or specific genetic subtypes. Notably, the majority of extramedullary relapses in our cohort involved in the testes, and these patients responded well to either chemotherapy or CAR-T therapy, consistent with previous reports (22, 23). Studies suggest that chemotherapy is effective for late isolated testicular relapse, while CAR-T therapy has emerged as a viable option for refractory or early relapsed testicular leukemia, contributing to improve outcomes (4). Our findings align with these observations, emphasizing the poor prognosis of bone marrow relapse and the potential benefit of HSCT in this subgroup. However, the role of HSCT requires careful consideration. In another study, while HSCT has been shown to improve disease-free survival (DFS) compared to chemotherapy (3-year DFS: 77.5 ± 6.2% vs. 66.9 ± 4.5%, *P* = 0.03), its effect on OS remains less definitive (24). Recent advances in bridging therapies, including multi-agent chemotherapy and immunotherapy such as blinatumomab, have shown considerable promise, particularly CAR-T therapy as a bridge to HSCT, which has been associated with improved outcomes in relapsed patients (25–28). Further research is warranted to establish the optimal bridging strategies prior to HSCT.

In conclusion, this study characterizes relapse patterns and prognostic determinants in pediatric ALL, highlighting the interplay among baseline thrombocytopenia, *BCR::ABL1* (+) fusion gene, and MRD level. Our findings substantiate HSCT as a cornerstone of bone marrow relapse management and suggest the potential integration of platelet recovery parameters into risk stratification algorithms These insights advocate for MRD-driven therapeutic intensification, and exploration of novel bridging regimens to enhance transplant outcomes. Future studies should focus on optimizing MRD monitoring schedules, mechanisms of thrombocytopenia-associated relapse, and personalized salvage therapies for high-risk subsets.

## Data Availability

The original contributions presented in the study are included in the article/Supplementary Material, further inquiries can be directed to the corresponding author.

## References

[1] Maloney KW Devidas M Wang C Mattano LA Friedmann AM Buckley P Outcome in children with standard-risk B-cell acute lymphoblastic leukemia. Results of children’s oncology group trial AALL0331. J Clin Oncol. (2020) 38(6):602–12. 10.1200/JCO.19.01086 31825704 PMC7030893

[2] Campbell M Kiss C Zimmermann M Riccheri C Kowalczyk J Felice MS Childhood acute lymphoblastic leukemia: results of the randomized acute lymphoblastic leukemia intercontinental-Berlin-Frankfurt-munster 2009 trial. J Clin Oncol. (2023) 41(19):3499–511. 10.1200/JCO.22.01760 37141547

[3] Angiolillo AL Schore RJ Kairalla JA Devidas M Rabin KR Zweidler-McKay P Excellent outcomes with reduced frequency of vincristine and dexamethasone pulses in standard-risk B-lymphoblastic leukemia: results from children’s oncology group AALL0932. J Clin Oncol. (2021) 39(13):1437–47. 10.1200/JCO.20.00494 33411585 PMC8274746

[4] Rheingold SR Bhojwani D Ji L Xu X Devidas M Kairalla JA Determinants of survival after first relapse of acute lymphoblastic leukemia: a children’s oncology group study. Leukemia. (2024) 38(11):2382–94. 10.1038/s41375-024-02395-4 39261601 PMC11518984

[5] Yang W Cai J Shen S Gao J Yu J Hu S Pulse therapy with vincristine and dexamethasone for childhood acute lymphoblastic leukaemia (CCCG-ALL-2015): an open-label, multicentre, randomised, phase 3, non-inferiority trial. Lancet Oncol. (2021) 22(9):1322–32. 10.1016/S1470-2045(21)00328-4 34329606 PMC8416799

[6] He J Munir F Catueno S Connors JS Gibson A Robusto L Biological markers of high-risk childhood acute lymphoblastic leukemia. Cancers (Basel). (2024) 16(5):858. 10.3390/cancers16050858 38473221 PMC10930495

[7] Conter V Bartram CR Valsecchi MG Schrauder A Panzer-Grumayer R Moricke A Molecular response to treatment redefines all prognostic factors in children and adolescents with B-cell precursor acute lymphoblastic leukemia: results in 3184 patients of the AIEOP-BFM ALL 2000 study. Blood. (2010) 115(16):3206–14. 10.1182/blood-2009-10-248146 20154213

[8] Vrooman LM Blonquist TM Harris MH Stevenson KE Place AE Hunt SK Refining risk classification in childhood B acute lymphoblastic leukemia: results of DFCI ALL consortium protocol 05-001. Blood Adv. (2018) 2(12):1449–58. 10.1182/bloodadvances.2018016584 29941458 PMC6020806

[9] Borowitz MJ Wood BL Devidas M Loh ML Raetz EA Salzer WL Prognostic significance of minimal residual disease in high risk B-ALL: a report from children’s oncology group study AALL0232. Blood. (2015) 126(8):964–71. 10.1182/blood-2015-03-633685 26124497 PMC4543229

[10] Popov A Henze G Tsaur G Budanov O Roumiantseva J Belevtsev M Flow cytometric minimal residual disease measurement accounting for cytogenetics in children with non-high-risk acute lymphoblastic leukemia treated according to the ALL-MB 2008 protocol. Cancer Med. (2024) 13(8):e7172. 10.1002/cam4.7172 38651186 PMC11036069

[11] Chen Y Liu R Li J. The significance of MRD-based strategy by dynamic assessment to guide treatment decisions in B-ALL - the enlightenment provided by demonstrating survival differences in the retrospective study. Hematology. (2024) 29(1):2415589. 10.1080/16078454.2024.2415589 39417653

[12] Pieters R de Groot-Kruseman H Van der Velden V Fiocco M van den Berg H de Bont E Successful therapy reduction and intensification for childhood acute lymphoblastic leukemia based on minimal residual disease monitoring: study ALL10 from the Dutch childhood oncology group. J Clin Oncol. (2016) 34(22):2591–601. 10.1200/JCO.2015.64.6364 27269950

[13] Eckert C Groeneveld-Krentz S Kirschner-Schwabe R Hagedorn N Chen-Santel C Bader P Improving stratification for children with late bone marrow B-cell acute lymphoblastic leukemia relapses with refined response classification and integration of genetics. J Clin Oncol. (2019) 37(36):3493–506. 10.1200/JCO.19.01694 31644328

[14] Chang TC Chen W Qu C Cheng Z Hedges D Elsayed A Genomic determinants of outcome in acute lymphoblastic leukemia. J Clin Oncol. (2024) 42(29):3491–503. 10.1200/JCO.23.02238 39121442 PMC11458106

[15] Shen S Chen X Cai J Yu J Gao J Hu S Effect of dasatinib vs imatinib in the treatment of pediatric Philadelphia chromosome-positive acute lymphoblastic leukemia: a randomized clinical trial. JAMA Oncol. (2020) 6(3):358–66. 10.1001/jamaoncol.2019.5868 31944221 PMC6990720

[16] Hunger SP Tran TH Saha V Devidas M Valsecchi MG Gastier-Foster JM Dasatinib with intensive chemotherapy in *de novo* paediatric Philadelphia chromosome-positive acute lymphoblastic leukaemia (CA180-372/COG AALL1122): a single-arm, multicentre, phase 2 trial. Lancet Haematol. (2023) 10(7):e510–20. 10.1016/S2352-3026(23)00088-1 37407142

[17] Dai Q Shi R Zhang G Yang H Wang Y Ye L Combined use of peripheral blood blast count and platelet count during and after induction therapy to predict prognosis in children with acute lymphoblastic leukemia. Medicine (Baltimore). (2021) 100(15):e25548. 10.1097/MD.0000000000025548 33847682 PMC8051997

[18] Ramesh R Aggarwal V Choudhary A Basu D Nair NS Ganesan P Peripheral blood neutrophil nadir and time to platelet recovery during induction chemotherapy: predictors of clinical outcomes and markers for optimizing induction treatment intensity in acute lymphoblastic leukemia. Asian Pac J Cancer Prev. (2024) 25(9):3229–37. 10.31557/APJCP.2024.25.9.3229 39342602 PMC11700331

[19] Zeidler L Zimmermann M Moricke A Meissner B Bartels D Tschan C Low platelet counts after induction therapy for childhood acute lymphoblastic leukemia are strongly associated with poor early response to treatment as measured by minimal residual disease and are prognostic for treatment outcome. Haematologica. (2012) 97(3):402–9. 10.3324/haematol.2011.045229 22058224 PMC3291595

[20] Donadieu J Auclerc MF Baruchel A Perel Y Bordigoni P Landman-Parker J Prognostic study of continuous variables (white blood cell count, peripheral blast cell count, haemoglobin level, platelet count and age) in childhood acute lymphoblastic leukaemia. Analysis of a population of 1545 children treated by the French acute lymphoblastic leukaemia group (FRALLE). Br J Cancer. (2000) 83(12):1617–22. 10.1054/bjoc.2000.1504 11104555 PMC2363446

[21] Jensen KS Oskarsson T Lahteenmaki PM Flaegstad T Jonsson OG Svenberg P Temporal changes in incidence of relapse and outcome after relapse of childhood acute lymphoblastic leukemia over three decades; a nordic population-based cohort study. Leukemia. (2022) 36(5):1274–82. 10.1038/s41375-022-01540-1 35314777

[22] Nguyen HTK Terao MA Green DM Pui CH. Inaba H: testicular involvement of acute lymphoblastic leukemia in children and adolescents: diagnosis, biology, and management. Cancer. (2021) 127(17):3067–81. 10.1002/cncr.33609 34031876 PMC9677247

[23] Chen X Wang Y Ruan M Li J Zhong M Li Z Treatment of testicular relapse of B-cell acute lymphoblastic leukemia with CD19-specific chimeric antigen receptor T cells. Clin Lymphoma Myeloma Leuk. (2020) 20(6):366–70. 10.1016/j.clml.2019.10.016 32205078 PMC8312220

[24] Lew G Chen Y Lu X Rheingold SR Whitlock JA Devidas M Outcomes after late bone marrow and very early central nervous system relapse of childhood B-acute lymphoblastic leukemia: a report from the children’s oncology group phase III study AALL0433. Haematologica. (2021) 106(1):46–55. 10.3324/haematol.2019.237230 32001530 PMC7776266

[25] Mengxuan S Fen Z Runming J. Novel treatments for pediatric relapsed or refractory acute B-cell lineage lymphoblastic leukemia: precision medicine era. Front Pediatr. (2022) 10:923419. 10.3389/fped.2022.923419 35813376 PMC9259965

[26] Hu L Charwudzi A Li Q Zhu W Tao Q Xiong S Anti-CD19 CAR-T cell therapy bridge to HSCT decreases the relapse rate and improves the long-term survival of R/R B-ALL patients: a systematic review and meta-analysis. Ann Hematol. (2021) 100(4):1003–12. 10.1007/s00277-021-04451-w 33587155

[27] Hu GH Zhao XY Zuo YX Chang YJ Suo P Wu J Unmanipulated haploidentical hematopoietic stem cell transplantation is an excellent option for children and young adult relapsed/refractory Philadelphia chromosome-negative B-cell acute lymphoblastic leukemia after CAR-T-cell therapy. Leukemia. (2021) 35(11):3092–100. 10.1038/s41375-021-01236-y 33824464

[28] Buechner J Caruana I Kunkele A Rives S Vettenranta K Bader P Chimeric antigen receptor T-cell therapy in paediatric B-cell precursor acute lymphoblastic leukaemia: curative treatment option or bridge to transplant? Front Pediatr. (2021) 9:784024. 10.3389/fped.2021.784024 35145941 PMC8823293

