# Supplementary material for: Pediatric acute lymphoblastic leukemia relapse and prognosis: key predictors and therapeutic implications
Source: Front Pediatr. 2025 Dec 18;13:1710578. doi: 10.3389/fped.2025.1710578 (PMC12756419; doi:10.3389/fped.2025.1710578)
Supplement: Supplementary file 1 [file Table1.docx]

**Supplemental Table S1. Treatment regimen of Chinese Children’s Cancer Group ALL-2015 protocol** [5]

The CCCG-ALL-2015 protocol is a prospective clinical research protocol. It is revised based on the ALL 2005 protocol of Shanghai Children's Medical Center (Shanghai Children's Medical Center acute lymphoblastic leukemia 2005 protocol, SCMC-2005-ALL) and the ST.JUDE Total and protocols of the St. Jude Children's Research Hospital . The main contents include: (1) Before the start of induction therapy, dexamethasone is administered for 4 days, and intrathecal injection and induction remission chemotherapy are performed on the 5th day. The chemotherapy intensity is different according to different risk levels. For example, if the MRD is > 1.00% on the 19th day of T-ALL or B-ALL, cyclophosphamide + cytarabine + 6-mercaptopurine is added once. (2) The number of intrathecal injections during the induction period and maintenance period varies for different clinical risk groups. The low-risk group has a total of 16 intrathecal injections, while the medium and high-risk groups have a total of 20 intrathecal injections. (3) The number of courses of high-dose methotrexate (HD-MTX) is reduced to 4 times. (4) The number of prednisolone injections is determined based on the clinical risk level. It is 3 times for the low-risk group and 9 times for the medium and high-risk groups. (5) Only the high-risk group has transplantation indications. After about 3 months of early treatment, they enter the allogeneic hematopoietic stem cell process. If transplantation is not accepted, the chemotherapy regimen is the same as that of the medium-risk group. (6) The treatment intensity is adjusted based on the MRD detection levels on the 19th day and 46th day of induction. (7) In medical centers with conditions, intravenous sedation is provided during the first intrathecal injection; the cerebrospinal fluid is sent for flow cytometric immunophenotyping analysis. (8) Radiotherapy for preventing central nervous system (CNS) leukemia is not used.

The Treatment regimen of Chinese Children’s Cancer Group ALL-2015 protocol is shown below.

1. **Remission induction and early intensification therapy**

| **Prephase treatment and remission induction** | | | | | | |
| --- | --- | --- | --- | --- | --- | --- |
| **Agents** | | **Doses and routes** | | | **Schedules** | |
| Dexamethasone | | 6 mg/m^2^/day IV or PO (b.i.d) | | | Days 1–4 (4 days) | |
|  |  | WBC ≥50 × 10^9^ /L, add | | | Days 0–4 (5 days) | |
|  |  | 3 mg/m^2^/day PO on Day 0 | | |  |  |
| Prednisone | | 45 mg/m^2^/day PO (t.i.d); | | | Days 5–28 (24 days) | |
|  |  | T-cell: 60 mg/m^2^/day PO | | |  |  |
| Vincristine | | 1∙5 mg/m^2^ IV (max 2 mg) | | | Days 5, 12, 19, 26 (4 doses) | |
| Daunorubicin | | 25 mg/m^2^ IV | | | Days 5, 12 (2 doses) | |
| L-asparaginase* | | 6000 U/m^2^ IM or IV | | | LR: alternate Days 6–24 (10 doses) | |
| Pegaspargase | | 2000 U/m^2^ IM | | | I/HR: Days 6, 26 (2 doses) | |
| Triple intrathecal^†^ | | Age-dependent | | | LR: Days 5, 19 | |
|  |  |  |  |  | IR: Days 5, 12, and 19 | |
|  |  |  |  |  | HR, T-ALL, CNS2, CNS3, and traumatic tap with blast: Days 5, 8, 12, 15, 19 | |
| Cyclophosphamide | | 1000 mg/m^2^ IV over 1 hour | | | Day 29 (1 dose) | |
| Cytarabine | | 50 mg/m^2^ SC, every 12 hours | | | Days 29–35 (14 doses) | |
| Mercaptopurine | | 60 mg/m^2^/day PO | | | Days 29–35 (7 doses) | |
| Triple intrathecal^†^ | | Age-dependent | | | Day 29 (1 dose) | |
| **T-ALL or B-ALL with Day 19 MRD ≥1%** | | | | | | |
| **Early intensification therapy** | | | | | | |
| **Agents** | | **Doses and Routes** | | | **Schedules** | |
| Vincristine | | 1∙5 mg/m^2^ IV (max 2 mg) | | | Days 50, 57 (2 doses) | |
| Pegaspargase | | 2000 U/m^2^ IM | | | Day 50 (1 dose) | |
| Cyclophosphamide | | 1000 mg/m^2^ IV over 1 hour | | | Day 50 (1 dose) | |
| Cytarabine | | 50 mg/m^2^ SC | | | Days 50–56 (14 doses) | |
|  |  | every 12 hours | | |  |  |
| Mercaptopurine | | 60 mg/m^2^/day PO | | | Days 50–56 (7 doses) | |
| Triple intrathecal^†^ | | Age-dependent | | | Day 50 (1 dose) | |
| *****L-asparaginase was replaced by pegaspargase at 2000 U/m^2^ IM on Day 6 in October 2017. | | | | | | |
| ^†^ **Triple intrathecal:** | | | | | | |
| Age <12 months: methotrexate 6 mg + cytarabine 15 mg + dexamethasone 2∙5 mg + normal saline 6 mL; | | | | | | |
| Age 12–36 months: methotrexate 9 mg + cytarabine 25 mg + dexamethasone 2∙5 mg + normal saline 6 mL; | | | | | | |
| Age ≥ 36 months: methotrexate 12∙5 mg + cytarabine 35 mg + dexamethasone 5 mg + normal saline 10 mL.  **Abbreviations:** I/HR, intermediate-/high-risk; IM, intramuscular; IV, intravenous; LR, low-risk; max, maximum; MRD, minimal residual disease; PO, by mouth; SC, subcutaneous; T-ALL, T-cell acute lymphoblastic leukemia; b.i.d., twice per day; t.i.d., three times a day; WBC, white blood cell | | | | | | |
|  |  | |  |  | |  |

1. **Consolidation therapy**

| **Agent** | **Doses and routes** | **Schedules** |
| --- | --- | --- |
| Methotrexate | LR:3 g/m^2^ IV; I/HR:5 g/m^2^ IV | Days 1, 15, 29, 43 (4 doses) |
| Mercaptopurine | 25 mg/m^2^ PO | Days 1–56 (56 doses) |
| Leucovorin | 15 mg/m^2^ IV | 3 doses at 42, 48, and 54 hours after the start of methotrexate and additional doses every 6 hours for patients with increased creatinine level and until serum methotrexate level <1 µmol/L |
|  | Adjusted according to  serum methotrexate level |  |
| Triple intrathecal | Age-dependent | Days 1, 15, 29, 43 (4 times) |

**Abbreviations:** I/HR, intermediate-/high-risk; IV, intravenous; LR, low-risk; PO, by mouth

1. **Continuation therapy**

| **Continuation and reinduction therapy (Weeks 16–53)** | | | | |
| --- | --- | --- | --- | --- |
| **Week** | **LR** |  | **I/HR** | |
| **16** | Dexamethasone + Vincristine + Mercaptopurine + IT |  | Dexamethasone + Vincristine + Mercaptopurine +Daunorubicin + Pegaspargase + IT | |
| **17** | Mercaptopurine + Methotrexate |  | Mercaptopurine |  |
| **18** | Mercaptopurine + Methotrexate |  | Mercaptopurine |  |
| **19** | Dexamethasone + Vincristine + Mercaptopurine + IT |  | Dexamethasone + Vincristine + Mercaptopurine +Daunorubicin + Pegaspargase + IT | |
| **20** | Mercaptopurine + Methotrexate |  | Mercaptopurine |  |
| **21** | Mercaptopurine + Methotrexate |  | Mercaptopurine |  |
| **22** | **Reinduction 1** |  | Dexamethasone + Vincristine + Mercaptopurine + Daunorubicin + Pegaspargase + IT | |
|  | Dexamethasone + Vincristine +   Daunorubicin + L-asparaginase* + IT |  |  |  |
| **23** | **Reinduction 1** |  | Mercaptopurine | |
|  | Vincristine |  |  |  |
| **24** | **Reinduction 1** |  | Mercaptopurine | |
|  | Dexamethasone + Vincristine |  |  |  |
| **25** | Mercaptopurine + Methotrexate | | Dexamethasone + Vincristine + Mercaptopurine + Daunorubicin + Pegaspargase + IT | |
| **26** | Mercaptopurine + Methotrexate |  | Mercaptopurine |  |
| **27** | Mercaptopurine + Methotrexate |  | Mercaptopurine |  |
| **28** | Dexamethasone + Vincristine + Mercaptopurine + IT |  | Dexamethasone + Vincristine + Mercaptopurine + Daunorubicin + Pegaspargase + IT | |
| **29** | Mercaptopurine + Methotrexate |  | Mercaptopurine |  |
| **30** | Mercaptopurine + Methotrexate |  | Mercaptopurine |  |
| **31** | Mercaptopurine + Methotrexate |  | Mercaptopurine |  |
| **32** | **Reinduction 2** |  | **Reinduction**  Dexamethasone + Vincristine + Cytarabine+ Pegaspargase + IT | |
|  | Dexamethasone + Vincristine +  L-asparaginase* + IT |  |  |  |
| **33** | **Reinduction 2** Vincristine |  | **Reinduction** Vincristine |  |
|  |  |  |  |  |
| **34** | **Reinduction 2** |  | **Reinduction** Dexamethasone + Vincristine | |
|  | Dexamethasone + Vincristine |  |  |  |
| **Following 4 weekly cycles for 5 cycles (Weeks 35–53)** | | | | |
| **35** | Mercaptopurine + Methotrexate | | Mercaptopurine + Methotrexate | |
| **36** | Mercaptopurine + Methotrexate | | Mercaptopurine + Methotrexate | |
| **37** | Mercaptopurine + Methotrexate | | Dexamethasone + Vincristine + Cyclophosphamide + Cytarabine + IT | |
| **38** | Dexamethasone + Vincristine + Mercaptopurine + IT (only in cycles 1 to 4) | | No chemotherapy | |
| *L-asparaginase was replaced by pegaspargase at 2000 U/m^2^ IM on Day 6 in October 2017.  Dexamethasone 8 mg/m^2^ for 7 days (LR) or 12 mg/m^2^ for 5 days (I/HR); vincristine (1∙5 mg/m^2^; max 2 mg); daunorubicin 25 mg/m^2^; pegaspargase 2000 U/m^2^ IM; mercaptopurine 50 mg/m^2^ for 7 days (25 mg/m^2^ during Weeks 16 and 31 for I/HR) methotrexate (25 mg/m^2^ PO on Day 1); cytarabine 300 mg/m^2^ IV; cyclophosphamide 300 mg/m^2^ IV. | | | | |
|  |  |  |  |  |

**Abbreviations:** I/HR, intermediate-/high-risk; IM, intramuscular; IT, intrathecal; IV, intravenous; LR, low-risk; PO, by mouth

1. **Reinduction therapy**

| **Risk group** | **Agents** |  | **Doses and Routes** | **Schedules** |
| --- | --- | --- | --- | --- |
| **LR Reinduction 1 and 2 (Weeks 7–9 and 17–19)** | | | | |
|  | Dexamethasone |  | 8 mg/m^2^/day IV/PO | Days 1–7, and Days 15–21 (14 days) |
|  | Vincristine |  | 1∙5 mg/m^2^ IV (max 2 mg) | Day 1, 8, 15 (3 doses) |
|  | Daunorubicin |  | 25 mg/m^2^ IV | Day 1 (1 dose) (Reinduction 1 only) |
|  | L- asparaginase* |  | 6000 U/m^2^ IV | Alternate Days 3–21 (10 doses) |
|  | Triple intrathecal |  | Age-dependent | Day 1 (1 dose) |
| **I/HR Reinduction (Weeks 17–19)** | | | | |
|  | Dexamethasone |  | 8 mg/m^2^/day IV/PO | Days 1–7, and Days15–21 (14 days) |
|  | Vincristine |  | 1∙5 mg/m^2^ IV (max 2 mg) | Days 1, 8, and 15 (3 doses) |
|  | Cytarabine |  | 2 g/m^2^ IV every 12 hours | Days 1 and 2 (4 doses) |
|  | Pegaspargase |  | 2000 U/m^2^ IM/IV | Day 3 (1 dose) |
|  | Triple intrathecal |  | Age dependent | Day 1 (1 dose) |
| *****L-asparaginase was replaced by pegaspargase at 2000 U/m^2^ IM on Day 6 in October 2017. | | | | |

**Abbreviations:** I/HR, intermediate-/high-risk; IM, intramuscular; IV, intravenous; LR, low-risk; max, maximum; PO, by mouth

1. **Randomization treatment**

| **Later continuation therapy (Weeks 54–125)** |
| --- |
| \| **Randomized groups** \| **LR-A** \| **LR-B** \| **I/HR-A** \| **I/HR-B** \| \| --- \| --- \| --- \| --- \| --- \| \| **54** \| Mercaptopurine + Methotrexate \| Mercaptopurine + Methotrexate \| Mercaptopurine + Methotrexate \| Mercaptopurine + Methotrexate \| \| **55** \| Mercaptopurine + Methotrexate \| Mercaptopurine + Methotrexate \| Mercaptopurine + Methotrexate \| Mercaptopurine + Methotrexate \| \| **56** \| Mercaptopurine + Methotrexate \| Mercaptopurine + Methotrexate \| Mercaptopurine + Methotrexate \| Mercaptopurine + Methotrexate \| \| **57** \| Mercaptopurine + Methotrexate \| Mercaptopurine + Methotrexate \| Mercaptopurine + Methotrexate \| Mercaptopurine + Methotrexate \| \| **58** \| Mercaptopurine + Methotrexate \| Mercaptopurine + Methotrexate \| Mercaptopurine + Methotrexate \| Mercaptopurine + Methotrexate \| \| **59** \| Mercaptopurine + Methotrexate \| Mercaptopurine + Methotrexate \| Mercaptopurine + Methotrexate \| Mercaptopurine + Methotrexate \| \| **60** \| Mercaptopurine + Methotrexate \| Mercaptopurine + Methotrexate \| Dexamethasone + Vincristine + Cyclophosphamide + Cytarabine \| Cyclophosphamide + Cytarabine \| \| **61** \| Dexamethasone + Vincristine + Mercaptopurine + Methotrexate \| Mercaptopurine + Methotrexate \| No chemotherapy \| No chemotherapy \| \| 8-weekly cycles (Weeks 54–109) \| \| \| \| \| \| Mercaptopurine + Methotrexate (Weeks 110–125) \| \| \| \| \| |

LR: dexamethasone 6 mg/m^2^ PO per day in two divided doses for 7 days; vincristine 1∙5 mg/m^2^ IV (max 2 mg) on Day 1 of the week; mercaptopurine 50 mg/m^2^ PO daily for 7 days per week; methotrexate 25 mg/m^2^ PO on Day 1 of the week.

I/HR: dexamethasone 6 mg/m^2^ PO per day in two divided doses for 7 days; vincristine 1∙5 mg/m^2^ IV (max 2 mg) on Day 1 of the week; mercaptopurine 50 mg/m^2^ PO daily for 7 days per week; methotrexate 25 mg/m^2^ PO on Day 1 of the week; cyclophosphamide 300 mg/m^2^ IV on Day 1 of the week; cytarabine 300 mg/m^2^ IV on Day 1 of the week.

**Abbreviations:** I/HR-A, intermediate-/high-risk group A; I/HR-B, intermediate-/high-risk group B; IT, intrathecal; IV, intravenous; LR-A, low-risk group A; LR-B, low-risk group B; max, maximum; PO by mouth
